# First records of the genera *Mariaplax* Rahayu & Ng, 2014 and *Hexapinus* Manning & Holthuis, 1981 from India, with descriptions of three new species (Crustacea, Decapoda, Brachyura, Hexapodidae)

**DOI:** 10.3897/zookeys.1292.204005

**Published:** 2026-09-23

**Authors:** Mani Prema, Sherine Sonia Cubelio, Peter K. L. Ng

**Affiliations:** 1 Centre for Marine Living Resources and Ecology, Ministry of Earth Sciences, Kochi – 682508, Kerala, India Centre for Marine Living Resources and Ecology, Ministry of Earth Sciences Kochi India; 2 Lee Kong Chian Natural History Museum, National University of Singapore, 2 Conservatory Drive, Singapore 117377, Singapore Lee Kong Chian Natural History Museum, National University of Singapore Singapore Singapore

**Keywords:** Biodiversity, Hexapodidae, Indian waters, new record, new species, six-legged crab, taxonomy

## Abstract

Three new brachyuran crab species of Hexapodidae are described from the Arabian Sea and Bay of Bengal, based on material collected by FORV Sagar Sampada between 2015 and 2019, with the genera *Mariaplax* Rahayu & Ng, 2014, and *Hexapinus* Manning & Holthuis, 1981, reported from Indian waters for the first time. *Mariaplax kochazhi***sp. nov**., and *M. albucus***sp. nov**. were collected from the Arabian Sea at a depth 30 m while *Hexapinus clivus***sp. nov**. was collected from the Bay of Bengal. *Mariaplax kochazhi***sp. nov**. is most similar to *M. granulifera* (Campbell & Stephensen, 1970) from Australia but can be distinguished by its proportionately wider carapace with the surfaces possessing larger granules, the pterygostomial region smooth with 5–7 striae, a rectangular ischium and more granulated merus of the third maxilliped, a subpentagonal male pleon telson, and a strongly curved S-shaped male first gonopod with a rounded distal tip. *Mariaplax albucus***sp. nov**. is similar to *M. sundaica* Rahayu & Widyastuti, 2018, from Java, but can easily be differentiated by a carapace that is as long as wide, a pterygostomial region that has 6 oblique striae, a relatively shorter and stouter P4 merus (4.0 times wider than long) and the distinctive structure of the vulva. In the latter species, the vulval operculum cover differs markedly from *M. kochazhi*, which is simple, rounded, and lacks a cover. *Hexapinus clivus***sp. nov**. is morphologically close to the widely distributed *H. simplex* Rahayu & Ng, 2014, from the West Pacific, but it can easily be differentiated by the weaker granulation on the carapace surface; possession of a relatively shorter and stouter P4 merus (2.5 times as long as wide); a proportionately wider male thoracic sternum, with the fused sternites 1 and 2 being subtriangular and the lateral margins gently sloping; and the semilunate vulva elevated and protruded, and positioned adjacent to the suture between sternites 5 and 6.

## Introduction

The brachyuran crabs of the family Hexapodidae Miers, 1886, in the Indo-West Pacific have been extensively revised, with 16 genera and 43 species currently recognised (Rahayu and Ng 2014; Ng and Rahayu 2015; Rahayu and Widyastuti 2018; Ng and Wong 2019; Naruse 2020; Poore and Ahyong 2023; Sato et al. 2025). Most of the recent taxa have been reported from the Pacific, with only a few reports from the Indian Ocean. India has records for only two genera with three species: *Lambdophallus sexpes*, Alcock, 1900, from the southwestern coast, *Hexapus sexpes* (Fabricius, 1798) from the Bay of Bengal, and *H. bidentatus* Velip & Rivonker, 2014 from the central-western coast of India (Trivedi et al. 2018).

The collection of brachyuran crabs of the Indian Exclusive Economic Zone (EEZ) through the systematic exploratory surveys of the Fishery Oceanographic Research Vessel (FORV) Sagar Sampada is stored in the Referral Centre at the Centre for Marine Living Resources and Ecology, Kochi. The collections included hexapodid specimens of three new species belonging to two genera, *Mariaplax* Rahayu & Ng, 2014 and *Hexapinus* Manning & Holthuis, 1981. Both genera are new records from Indian EEZ. The three new species, *Mariaplax kochazhi* sp. nov., *M. albucus* sp. nov. and *Hexapinus clivus* sp. nov., are described and compared with allied taxa.

## Material and methods

The specimens were collected from the Arabian Sea during cruises 343 and 392 by the FORV Sagar Sampada and were preserved in 5% formaldehyde before being transferred to 70% alcohol. The photographs were taken with a Leica S9i microscope, the illustrations were sketched using vector graphics in Adobe Illustrator CS2 and edited in Adobe Photoshop. The specimens used for Scanning Electron Microscopy (SEM) were cleaned manually with a soft brush in 70% alcohol, dehydrated in an ascending series of ethanol and air-dried overnight in a desiccator. The processed specimen was mounted on silver stubs using Leit adhesive carbon tabs (12 mm). SEM micrographs were taken using an environmental SEM ZEISS EVO 15 with an extended-range cascade current detector (C2DX), set at 10 kV. The specimen was not gold-coated and, after analysis, was preserved in 90% alcohol and deposited in the museum.

Specimens examined are deposited in the Bhavasagara Referral Centre: National Repository for Reference Collections of Deep-Sea Fauna at Centre for Marine Living Resources and Ecology, Kochi (**CMLRE**); with comparative material used retained in the Zoological Reference Collection of the Lee Kong Chian Natural History Museum (**ZRC**), National University of Singapore. Details of all specimens examined are available in the Material examined subsection of the systematic accounts below. Measurements provided, in millimetres (**mm**), are of the maximum carapace width and length (taken at the midline from the tips of the frontal margin to the median part of the posterior margin), respectively. The terminology used follows Rahayu and Ng (2014). Abbreviations used in this study are as follows: **coll. =** collector; **juv. =** juvenile; **G1** = male first gonopod; **G2** = male second gonopod; **P2–P4** = pereopods 2–4 (first to third ambulatory legs), respectively.

## Systematic account

### Superfamily Hexapodoidea Miers, 1886

**Family Hexapodidae Miers, 1886**

#### Genus Mariaplax

Taxon classificationAnimaliaDecapodaHexapodidae

Rahayu & Ng, 2014

1C14583F-D80F-5FB4-9A5F-1EAD3A9A2839

##### Type species.

*Lambdophallus anfractus* Rathbun, 1909; type by original designation.

##### Remarks.

The genus *Mariaplax* is currently represented by 19 species, all from the Indo-West Pacific (cf. Rahayu and Ng 2014; Ng and Rahayu 2015; Rahayu and Widyastuti 2018; Ng and Wong 2019). The relatively wide and granular carapaces of the two new species, *M. kochazhi* sp. nov. and *M. albucus* sp. nov. (1.5–1.8 times longer than wide) ally them with *M. chenae* Rahayu & Ng, 2014, *M. narusei* Rahayu & Ng, 2014, *M. propinqua* Rahayu & Ng, 2014, *M. secus* Rahayu & Ng, 2014, *M. hispida* Rahayu & Widyastuti, 2018, and *M. sundaica* Rahayu & Widyastuti, 2018, all of which have carapaces that are 1.5 times or more longer than wide (see Rahayu and Ng 2014; Rahayu and Widyastuti 2018). In general, the appearance of *M. albucus* sp. nov., with its wider rectangular carapace with granulation pattern, third maxilliped, and sternopleonal cavity, resembles *M. sundaica*, which was originally described from Selat Sunda, Indonesia (Rahayu and Widyastuti 2018); while *M. albucus* sp. nov. is closer to *M. kochazhi* sp. nov. by its wider, densely tuberculated carapace and wider sternopleonal cavity. Detailed comparisons of the two new species with congeners are done under the Remarks section for each species.

Rahayu and Ng (2014: 442) tentatively assigned the record of “*Hexapus* ? *granuliferus*” from the Andaman Sea by Serène and Soh (1976) to it because of the shapes of the third maxilliped and female pleon, but it needs to be verified because this record is some distance from the type locality, Queensland, Australia. It is possible that this record is actually *M. anfracta* (Rathbun, 1909) as there is a record of this species from nearby Penang (Rahayu and Ng 2014: 424), conspecific with one of the two new species of *Mariaplax* described here, or even an undescribed taxon.

#### Mariaplax kochazhi sp. nov.

Taxon classificationAnimaliaDecapodaHexapodidae

3AA92146-A08D-55E5-A4DB-3C26B62FC675

https://zoobank.org/A2EFED37-CAB7-40EF-A3DA-51F297FCDB94

**Figure 1.**
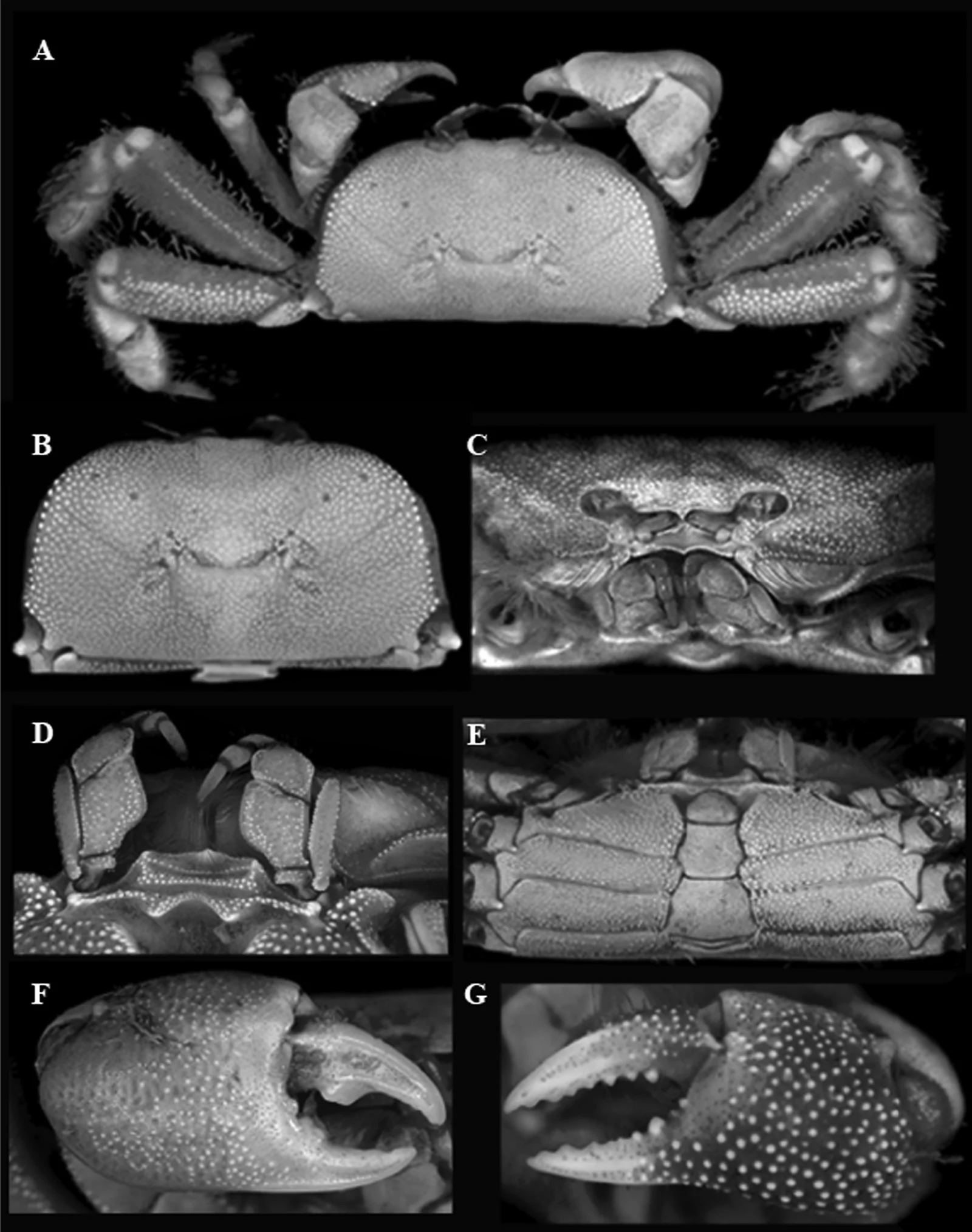
*Mariaplax kochazhi* sp. nov., holotype male, male (5.5 × 3.5 mm) (CMLRE-BC-BRY-1), Arabian Sea, India. **A**. Overall dorsal habitus; **B**. Dorsal view of carapace; **C**. Frontal view of cephalothorax showing third maxillipeds and pterygostomial region; **D**. Third maxillipeds and anterior thoracic sternum; **E**. Thoracic sternum and pleon; **F**. Outer view of right major chela; **G**. Outer view of left minor chela.

**Figure 2.**
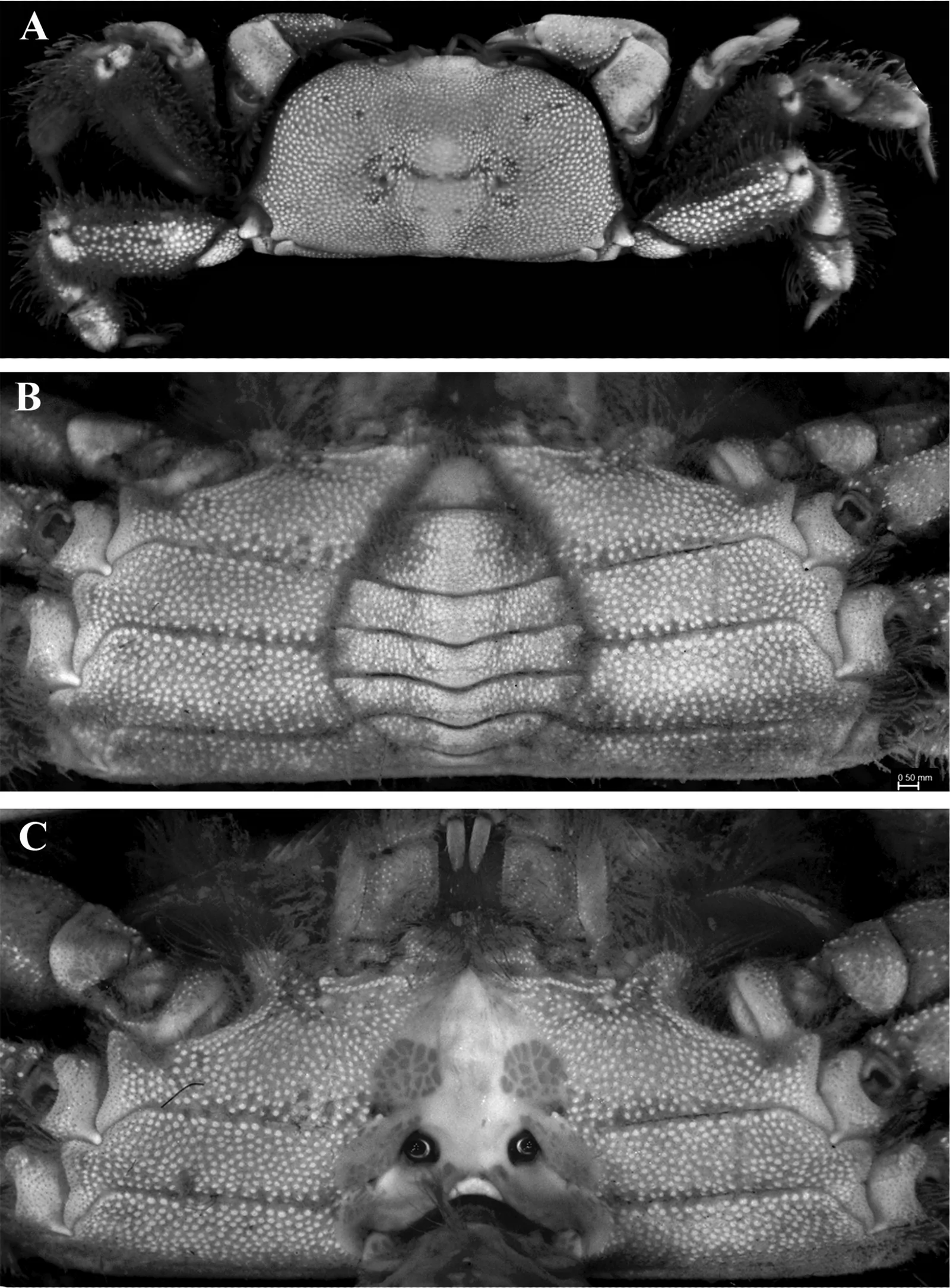
*Mariaplax kochazhi* sp. nov., paratype female (5.3 × 2.8 mm) (CMLRE-BC-BRY-2), Arabian Sea, India. **A**. Overall dorsal habitus; **B**. Thoracic sternum and pleon; **C**. Thoracic sternum showing vulvae.

**Figure 3.**
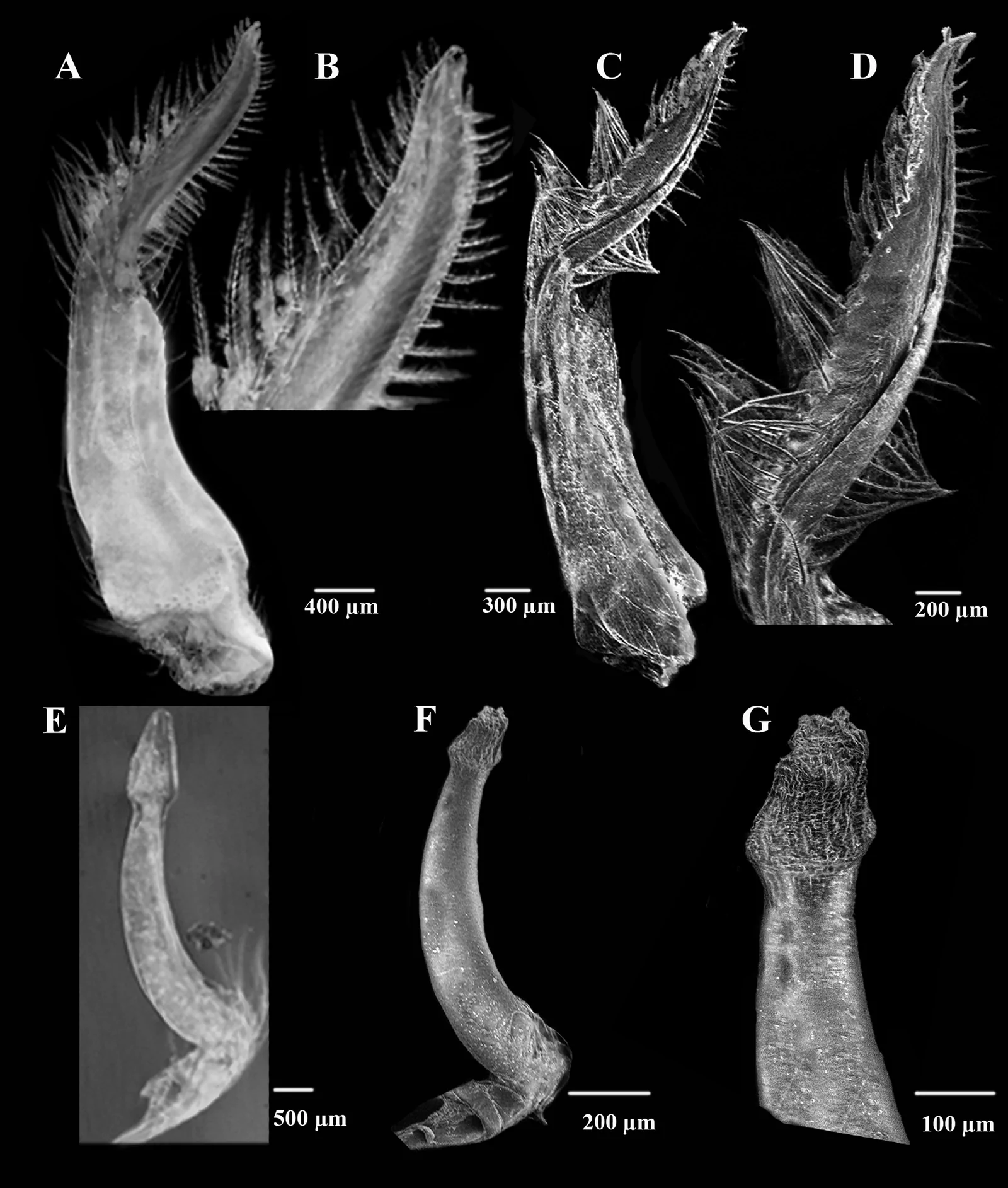
Microscopic and SEM micrographs of gonopods of *Mariaplax kochazhi* sp. nov., Arabian Sea, India. **A, B, E**. Holotype male (5.5 × 3.5 mm) (CMLRE-BC-BRY-1); **C, D, F, G**. Paratype male (6.7 × 4.0 mm) (CMLRE-BC-BRY-2), Arabian Sea, India. **A, C**. Left G1; **B–D**. Distal tip of the left G1; **E–G**. Left G2.

**Figure 4.**
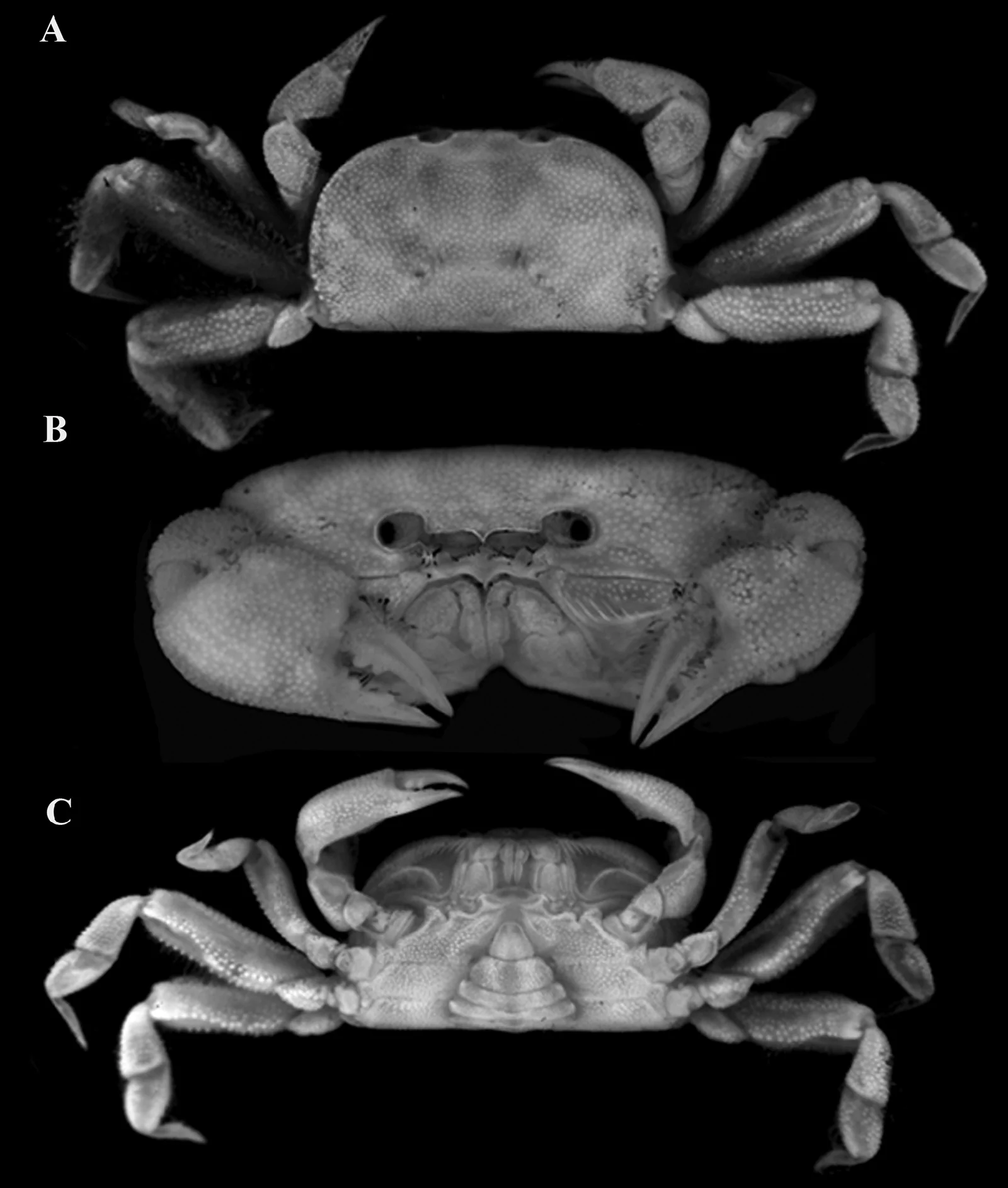
*Mariaplax albucus* sp. nov., holotype female (6.2 × 3.5 mm) (CMLRE-BC-BRY-6), Arabian Sea, India. **A**. Dorsal view of entire animal; **B**. Frontal view of cephalothorax showing third maxillipeds and pterygostomial region; **C**. Ventral view of cephalothorax.

Figs 1, 2, 3, 4, 6A, D, G, H, K–N, 7A–C, 8A–C

##### Material examined.

***Holotype***. • 1 male (5.5 × 3.5 mm) (CMLRE-BC-BRY-1), station 24, cruise number 343, grab, 30 m, sandy bottom, off Kochi, Arabian Sea, 9°57'54.0"N, 75°35'31.2"E, 16 August 2015. ***Paratypes***. • 1 female (5.3 × 2.8 mm) (CMLRE-BC-BRY-2), 1 male (6.7 × 4.0 mm) (CMLRE-BC-BRY-3), same data as holotype. ***Non-types***. • 6 females (10.1 × 5.7 mm, 6.6 × 4.1 mm, 5.9 × 3.0 mm, 4.9 × 2.8 mm, 4.5 × 2.6 mm, 4.0 × 2.1 mm), (CMLRE-BC-BRY-4) 1 male (5.0 × 3.1 mm) (CMLRE-BC-BRY-5) same data as holotype (all very fragile so not suitable to be type material).

##### Diagnosis.

Carapace subtrapezoidal, 1.5–1.7 times as wide as long, dorsal surface granular; pterygostomial region smooth with 5–7 striae; third maxilliped ischium rectangular, merus granulated; telson of male pleon subpentagonal; female pleon telson rounded; G1 strongly curved, S-shaped, distal tip rounded; vulvae simple, rounded, fully exposed, operculum absent.

##### Description.

Carapace subtrapezoidal, about 1.5–1.7 times as wide as long, dorsal surface densely granulated, anterolateral margin lined with distinct rounded granules; regions indistinct, median H-shaped elongated furrow towards anterolateral margin visible (Figs 1A, 1B, 2A, 6A, 8A). Each lobe of frontal margin with gently convex margin; antero-posterolateral margins arcuate, armed with larger tubercles; posterolateral margin with corner prominently protruding, forming distinct angle before strongly curved towards posterior margin (Fig. 1B); eye small, slightly movable, globular cornea, peduncle granular (Fig. 1C). Pterygostomial region generally smooth, with 5–7 oblique, long striae, upper margin with scattered granules; epistome longer, more pointed medially, shallowly bilobed lateral margins (Figs 1C, 8B). Third maxillipeds as long as wide, almost covering buccal cavity (Figs 1C, 1D, 6D, 8B); ischium of third maxillipeds distinctly wide, longer than merus, mesial margin straight; combined length of dactylus, propodus, and carpus relatively shorter than that of merus and ischium; exopod relatively narrow, flagellum well developed. Chelipeds stout, unequal (Fig. 1F, G); major chela (Fig. 1F) forming gap when fingers closed; dactylus and fixed finger with longitudinal groove on outer surface; palm slightly longer than wide, covered by sparse granules; minor chela (Fig. 1G) with small gap between closed fingers, dactylus and fixed finger with shallow, wider, longitudinal groove, palm more or less equally wide to its length, covered by densely packed, prominent, larger granules. P2–P4 (Figs 1A, 2A, 8C) relatively short, slender; deep longitudinal groove on merus with granular outer surface; P3 merus outer surface distinctly separated by granules, carpus and propodus smooth; P4 merus, carpus, propodus densely packed granules, with dactylus gently upcurved.

**Male** thoracic sternum wide (Fig. 1E), covered by dense tubercles; sternites 1 and 2 fused, separated from sternite 3 by distinct ridge, sternal spine for locking pleon present; thoracic sternal groove (anterolateral extension of sterno-pleonal cavity to accommodate G1) wide, deep, extends from sternopleonal cavity to base of sternite 3 (Fig. 1D, E); sternite 4 wide, sternopleonal cavity reaching distal margin of sternites 3. Male pleon relatively wide (Figs 1E, 6G), somites 1 and 2 free, somites 3–5 completely fused, wider than somite 6, lateral margin slightly convex to sinuous, somite 6 pentagonal, shorter than length of somites 3–5, lateral margin subproximally convex to blunt triangular projection; telson lateral margin slightly sinuous, lined with larger granules, distolateral margin gently concave, forming subpentagonal structure, setose densely.

G1 (Figs 3A–D, 6K–M), curved outwards medially at 60–70°, basal part stout, gently curved outwards, outer margin smooth, inner margin slightly serrated, curved; distal half slender, margin covered by straight, long and short plumose setae; distal tip broad, rounded, directed upwards, apex not concealed under closed pleon, exposed on thoracic sternal groove, obscured by setae of telson. G2 (Figs 3E–G, 6N) small, slightly curved, ridge separates distal region and tip.

**Female** thoracic sternum (Figs 2B, 2C, 7A–C), wide, densely covered by tubercles or rounded granules, sternites 1 and 2 fused, separated from sternite 3 by distinct ridge; sternopleonal cavity wide, smooth, reached to sternite 3; thoracic sutures 4/5 and 5/6 parallel and directed slightly posteromesially outside sternopleonal cavity, then bent anteromesially in sternopleonal cavity. Vulvae simple, rounded and completely exposed, operculum absent, located adjacent to mesial end of sutures 4/5 and 5/6, which is level with middle of sternite 5 outside cavity, pleonal locking spine present, on sternite 5 (Figs 2C, 7A–C); female pleon with 6 free somites and telson with rounded distal margin (Figs 2B, 6H).

##### Variation.

No obvious non-sexual differences exist between the sexes.

##### Colour.

Unknown.

##### Etymology.

The species is named after its type locality, off the coast of Kochi, using the original Malayalam form. The name *Kochi* is derived from the Malayalam word കൊച്ചുഴി or “*Koch-azhi*,” meaning “small sea” (*kochu* = small; *azhi* = sea). Kochi (formerly Cochin), a significant port city historically known as the “Queen of the Arabian Sea,” lies along the southwestern coast of India. The name is treated as a noun in apposition.

##### Type locality.

Off Kochi, Kerala, Arabian Sea.

##### Distribution.

Presently known only from the type locality.

##### Remarks.

The general appearance of *Mariaplax kochazhi* sp. nov., with its carapace shape, distinctly granulated surface and structure of the G1, allies it most closely with *M. granulifera* Campbell & Stephenson, 1970, originally described from Queensland, Australia (Campbell and Stephenson 1970; Rahayu and Ng 2014). *Mariaplax kochazhi* sp. nov. however, differs in that the carapace is slightly wider (1.5–1.7 times), with relatively larger granules on the dorsal surface and the median H-shaped depression deep with a more elongated groove (Figs 1A, 1B, 2A, 6A) (versus carapace relatively narrower, 1.3 times as wide as long, more quadrate, with relatively smaller granules and the H-shaped median depression less prominent without an elongated groove in *M. granulifera*; see Rahayu and Ng 2014: figs 34A, 35A); the ischium of the third maxilliped is slightly longer (0.7 times width) and more rectangular in shape, with more densely packed larger granules on the margins (Figs 1D, 6D) (versus ischium relatively shorter (0.5 times width), more quadrate in shape with only sparsely distributed granules on the margins in *M. granulifera*; see Rahayu and Ng 2014: figs 35C, 36E); the distolateral margin of the male telson is gently concave with the structure appearing subpentagonal (Fig. 6G) (versus clearly rounded in *M. granulifera*; see Rahayu and Ng 2014: fig. 36D); and the distal tip of the G1 is clearly more rounded with the basal part proportionately stouter and the outer margin more strongly curved (Figs 3A–D, 6K–M) (versus part more pointed, with the basal part of the G1 more slender and outer margin less curved in *M. granulifera*; see Rahayu and Ng 2014: fig. 36I, J).

In the proportions of the carapace, *M. kochazhi* sp. nov. is also similar to *M. hispida*, *M. propinqua*, and *M. chenae*, but they are very different in other characters. In *M. hispida*, the carapace is subquadrate in shape, the third maxilliped ischium is proportionately longer, and the vulva is semilunate in shape (see Rahayu and Widyastuti 2018: fig. 6A–E) (carapace subtrapezoidal, third maxilliped ischium shorter and vulva rounded in *M. kochazhi* sp. nov.; Figs 1A–D, 2C, 6A, 6D, 7A–C). In *M. propinqua*, the carapace is distinctly more tuberculate, the pterygostomial region has four oblique short striae and the upper margin has scattered granules, and the G1 is S-shaped with a tapered distal part and medially, there are strong three large teeth present (see Rahayu and Ng 2014: figs 43, 45 I, J) (carapace relatively less tuberculate, the pterygostomial region has 5–7 long oblique striae with the upper margin covered with scattered granules, (Fig. 1C); and the G1 is curved outwards medially with the distal tip broad and rounded, without teeth on the medial margin in *M. kochazhi* sp. nov.; Figs 3A–D, 6K–M). In *M. chenae*, the carapace is subquadrate in shape, the pterygostomial region has four oblique short striae with fewer granules on the upper margins, the G1 is S-shaped distally, with a tapered distal part and there is a row spines medially (see Rahayu and Ng 2014: figs 25A, B, I, J) (versus carapace subtrapezoidal, the pterygostomial region has 5–7 long oblique striae with the upper margin covered with scattered granules, and the G1 is curved outwards medially with the distal tip broad and rounded, without teeth on the medial margin in *M. kochazhi* sp. nov.; Figs 1, 3, 6K–M).

The vulva of *M. kochazhi* sp. nov. (Figs 2C, 7A–C), being slightly bulging, gently sloping mesially, somewhat subtriangular in form and lacking an operculum, with the two vulvae well separated and positioned adjacent to the mesial end of sutures 4/5 and 5/6 and level to the middle part of sternite 5, is similar in structure to that of *M. chenae* (see Rahayu and Widyastuti 2018: fig. 4D–F). In this aspect, its vulva is also similar to *M. pitrai* Rahayu & Widyastuti, 2018, although that of the latter is relatively larger and more subcircular vulvae in shape (see Rahayu and Widyastuti 2018: fig. 4C). The vulvae of *M. hispida*, *M. propinqua*, *M. chenae* and *M. pitrai*, however, are not bulging, with the surfaces not sloping mesially between sternites 6–8 in the sternopleonal cavity.

#### Mariaplax albucus sp. nov.

Taxon classificationAnimaliaDecapodaHexapodidae

F4BEE1FB-1AF0-5682-B49F-B24D2A177FA3

https://zoobank.org/900CDE67-2793-41BF-A1FE-AEB6868A2D8A

**Figure 5.**
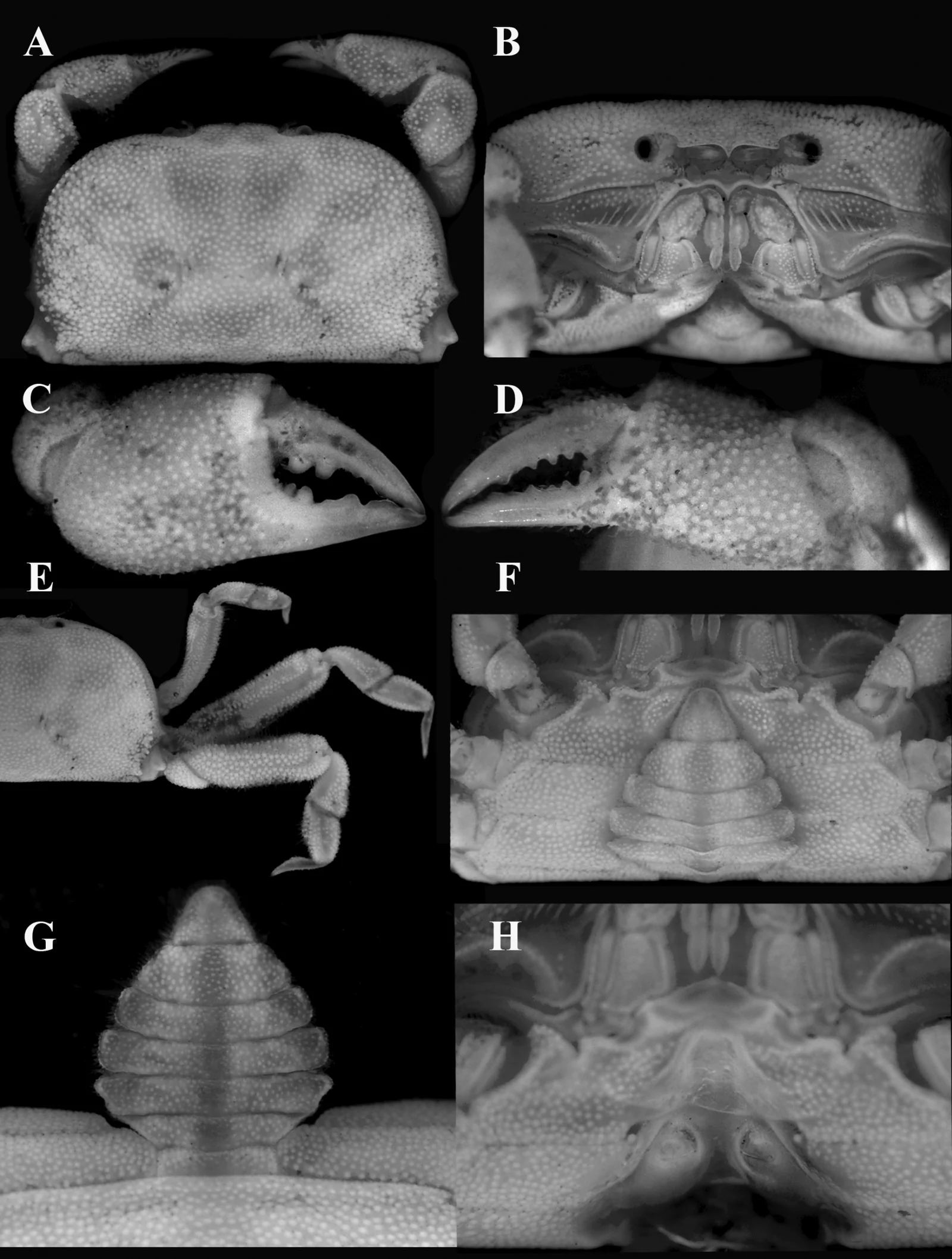
*Mariaplax albucus* sp. nov., holotype female (6.2 × 3.5 mm) (CMLRE-BC-BRY-6). **A**. Dorsal view of carapace; **B**. Frontal view of cephalothorax showing third maxillipeds and pterygostomial region; **C**. Outer view of right chela; **D**. Outer view of left chela; **E**. P2–P4; **F**. Thoracic sternum with pleon; **G**. Pleon; **H**. Thoracic sternum showing vulvae.

**Figure 6.**
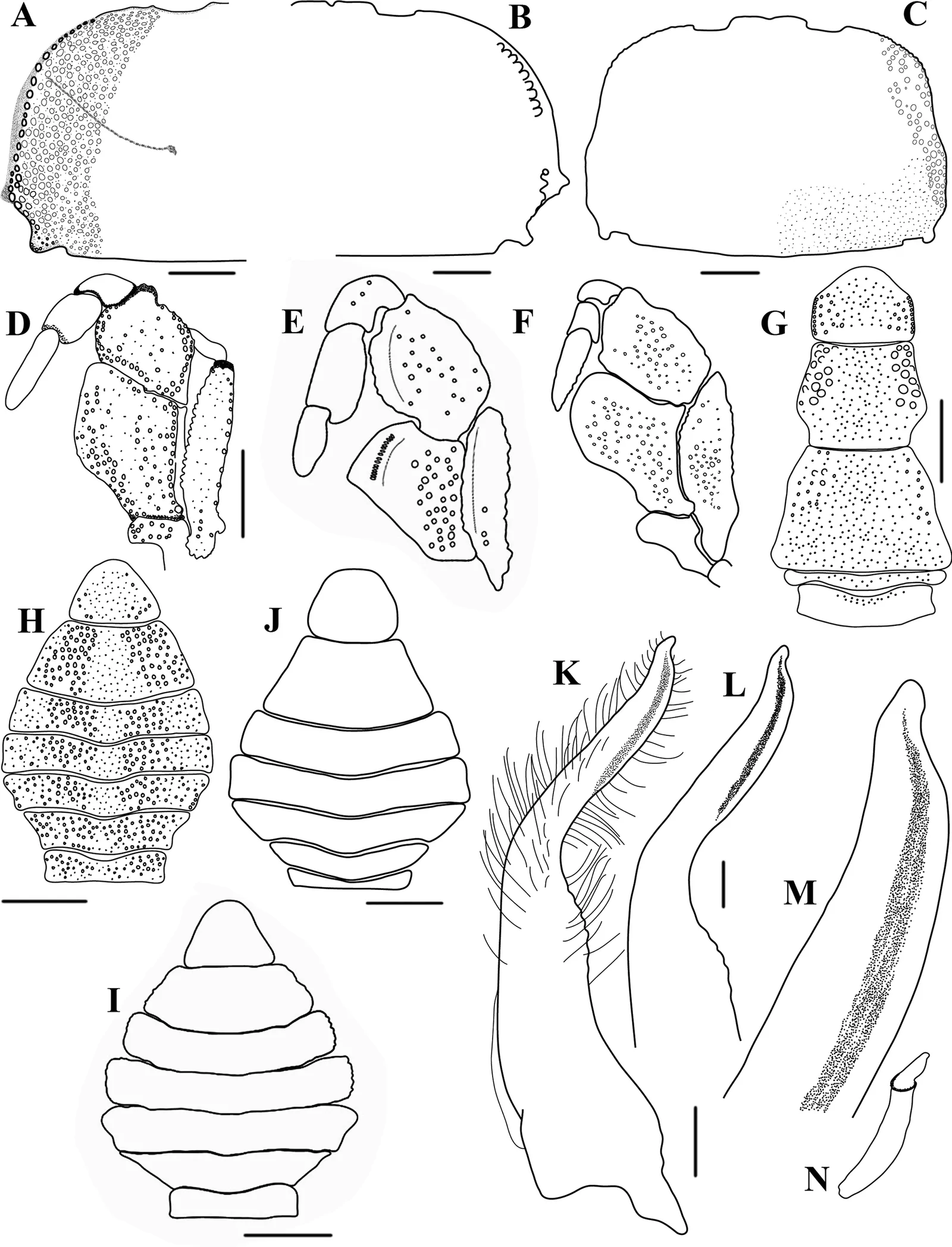
**A, D, G, H, K–N**. *Mariaplax kochazhi* sp. nov., holotype male, male (5.5 × 3.5 mm) (CMLRE-BC-BRY-1), Arabian Sea, India; **B, E, I**. *M. albucus* sp. nov., holotype female (6.2 × 3.5 mm) (CMLRE-BC-BRY-6); **C, F, J**. *Hexapinus clivus* sp. nov., holotype female (8.8 × 5.6 mm) (CMLRE-BC-BRY-8); **A–C**. Carapace; **D–F**. Third maxilliped; **G**. Male pleon; **H–J**. Female pleon; **K**. Left G1; **L**. Distal part of left G1 (setae not drawn); **M**. Distal most part of left G1 (setae not drawn); **N**. Left G2. **Scale bars**: 2.5 mm (**A, C**); 1.0 mm (**B, D–J**); 500 µm (**K**); 300 µm (**L–N**).

**Figure 7.**
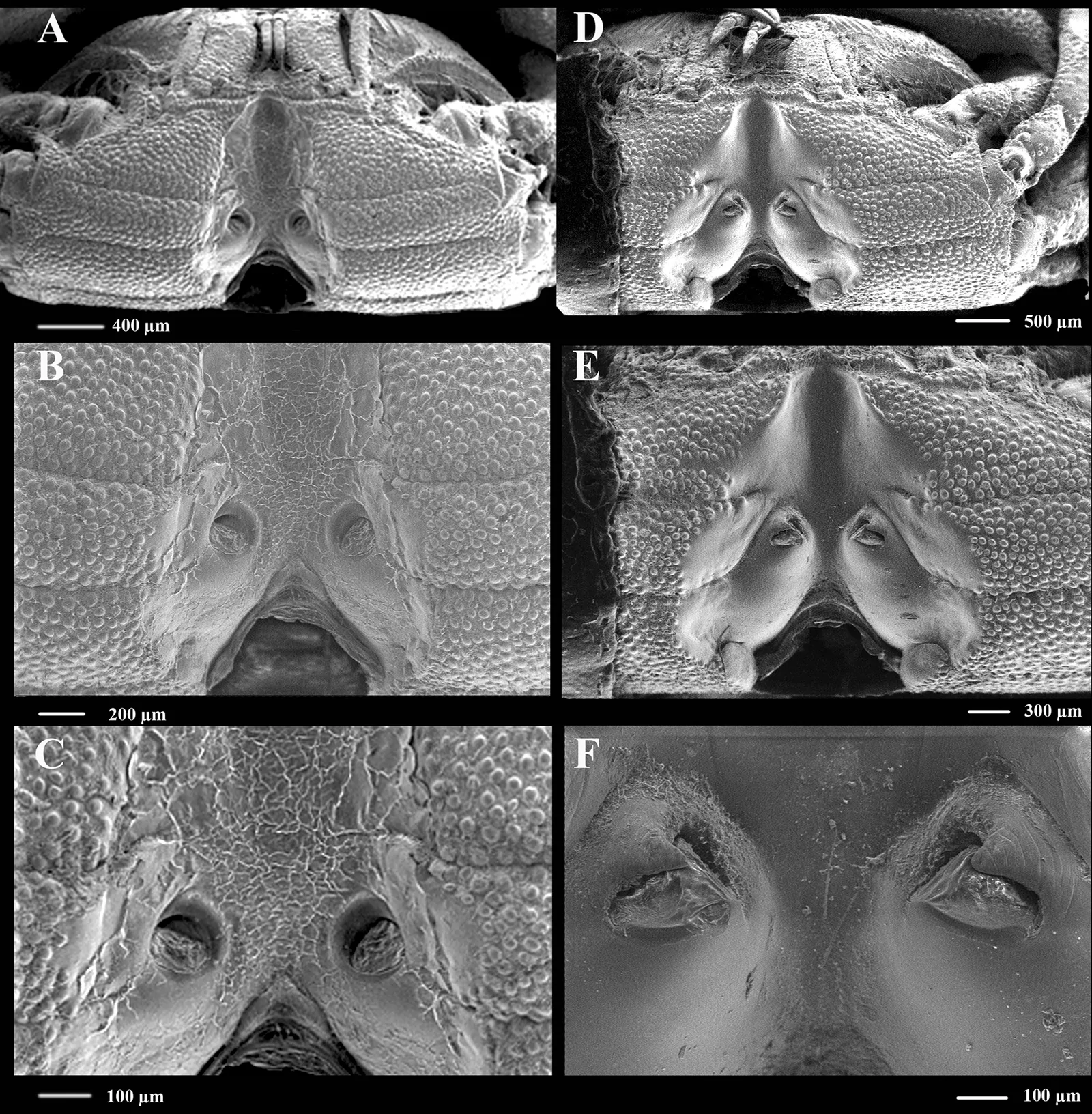
SEM micrographs of female thoracic sternum and vulvae at different magnifications. **A–C**. *Mariaplax kochazhi* sp. nov., paratype female (5.3 × 2.8 mm) (CMLRE-BC-BRY-2), Arabian Sea, India; **D–F**. *M. albucus* sp. nov., paratype female, (5.8 × 3.2 mm) (CMLRE-BC-BRY-7), Arabian Sea, India; **A, D**. Overall view of sternopleonal cavity; **B, E**. Close-up of sternites 4**–**6 and vulvae; **C, F**. Close-up view of both vulvae.

**Figure 8.**
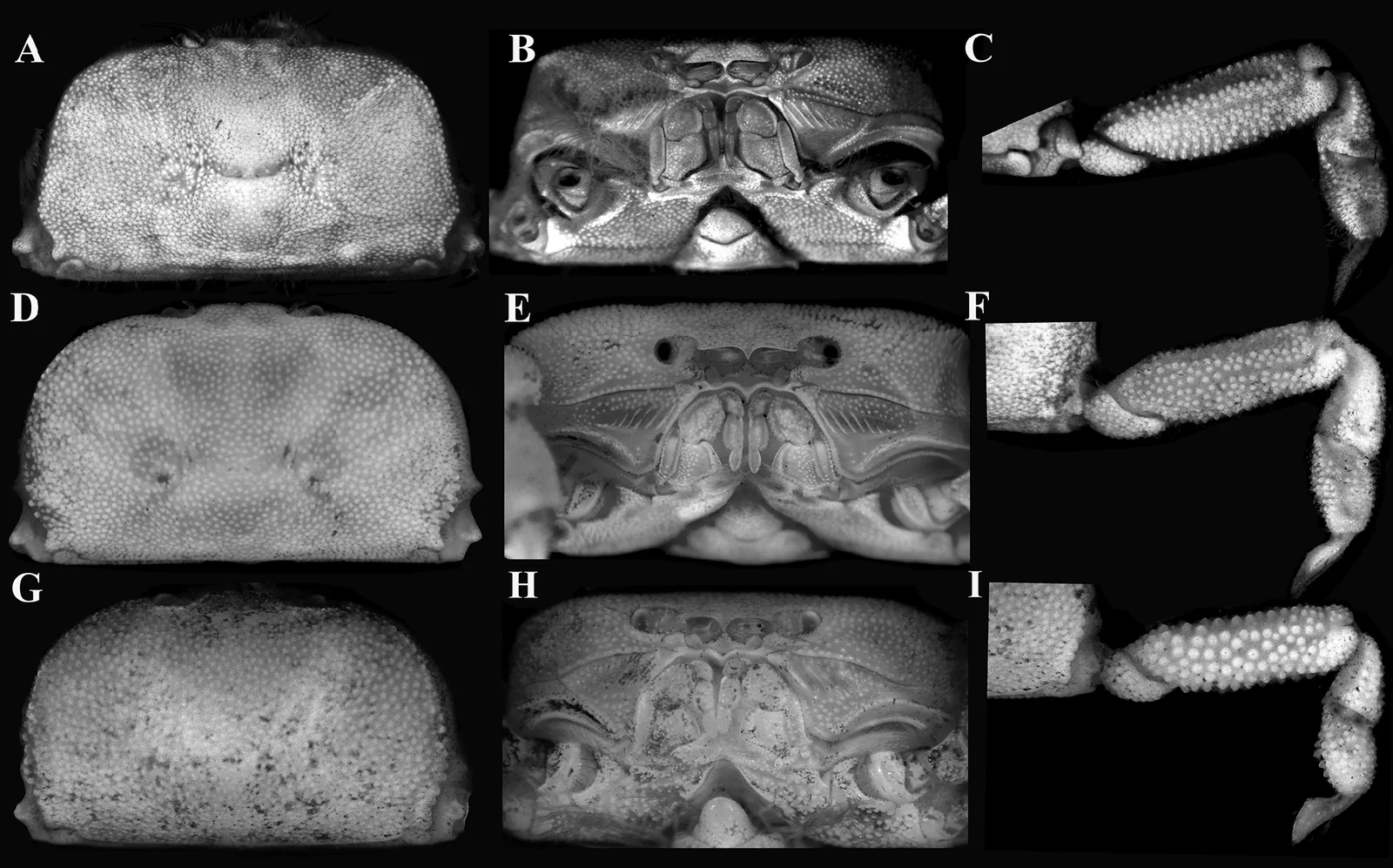
**A, B, C**. *Mariaplax kochazhi* sp. nov., holotype male (5.5 × 3.5 mm) (CMLRE-BC-BRY-1), Arabian Sea; **D, E, F**. *M. albucus* sp. nov., holotype female (6.2 × 3.5 mm) (CMLRE-BC-BRY-6) Arabian Sea; **G, H, I**. *M. sundaica* Rahayu & Widyastuti, 2018, female (4.4 × 2.5 mm) (ZRC 2018.0988), Java; **A, D, G**. Dorsal view of carapace; **B, E, H**. Frontal view of cephalothorax showing third maxillipeds and pterygostomial region; **C, F, I**. Right P4, dorsal view.

Figs 4, 5, 6 B, E, J, 7D–F, 8D–F

##### Material examined.

***Holotype***. • 1 female (6.2 × 3.5 mm) (CMLRE-BC-BRY-6) station 24, cruise number 343, grab, 30 m, sandy bottom, off Kochi, Arabian Sea, 9°57'54.0"N, 75°35'31.2"E, 16 August 2015. ***Paratypes***. • 2 females (5.8 × 3.2 mm, 6.3 × 3.5 mm, both fragile) (CMLRE-BC-BRY-7), same data as holotype.

##### Comparative material.

***Mariaplax sundaica* Rahayu & Widyastuti, 2018**: • 1 female (4.4 × 2.5 mm) (ZRC 2018.0988) station DW06, on mud and clay, 266–294 m, 5°43.413'S, 104°49.712'E – 5°43.779'S, 104°50.060'E, Sunda Strait (between Tabuan Island and Sumatra), Java, coll. SJADES 2018 cruise, *Baruna Jaya VIII*, 25 March 2018; • 1 female (3.8 × 2.3 mm), (ZRC 2018.0989), on mud, station DW01, 100–104 m, 6°20.489'S, 105°26.890'E – 6°20.431'S, 105°26.214'E, Sunda Strait (South of Krakatau Island), Java, coll. SJADES 2018 cruise, *Baruna Jaya VIII*, 25 March 2018; • 1 female (3.8 × 2.4 mm) (ZRC 2020.0398), station DW06, on mud and clay, Sunda Strait, between Tabuan Island and Sumatra, Java, Indonesia, Indian Ocean, 5°43.413'S, 104°49.712'E – 5°43.779'S, 104°50.060'E, 266–294 m, coll. SJADES 2018 cruise, *Baruna Jaya VIII*, 25 March 2018.

##### Diagnosis.

Carapace as wide as long, dorsal surface strongly granulated, pterygostomial with 6 oblique striae, P4 merus short and stout, vulvae large with operculum cover, located distally on bulged, sloped margin between sternites 6–8.

##### Description.

Carapace subquadrate, ca. 1.8 times as wide as long, dorsal surface strongly granulated; regions indistinct, with shallow grooves on branchial region (Figs 4A–C, 5A, 6B, 8D). Eyes small, slightly movable, cornea black, equal in size to granular peduncle (Fig. 5B). Pterygostomial regions (Figs 4B, 5B, 8E) relatively smooth, covered by granules, with 6 oblique striae. Epistome longer, pointed distally, with bilobed lateral margin (Figs 5B, 8E). Third maxillipeds wide (Figs 5B, 6E, 8E); merus slightly rectangular; ischium longer than merus, more pronounced subproximally, with oblique mesial margin, combined length of carpus, propodus and dactylus, shorter than merus and ischium; exopod relatively longer, stouter, granulated margins, flagellum well developed. Chelipeds asymmetrical, covered by dense tubercles with fringe of setae (Figs 4B, 5C, 5D); major chela (Fig. 5C) with gaping finger when closed, cutting edge at third proximal with 2 larger teeth; both dactyli with longitudinal groove medially, almost reaching proximal part but not to tip, tubercles on proximal third; palm as long as wide; outer surface of palm covered with closely-spaced tubercles; carpus outer surface with closely-spaced granules with denticulate mesial margin; merus short, covered with granules; minor chela (Fig. 5D) with relatively narrower gap when fingers closed; both dactyli with longitudinal ridge medially, not reaching proximal part, cutting edges of dactylus and fixed finger with row of small teeth, outer surface of palm, carpus and merus covered by closely-spaced tubercles; fringe of setae on each upper inner margin of merus. P2– P4 (Figs 5E, 8F) relatively long, slender, covered by dense tubercles, dactylus gently upcurved; P3 longest; dactylus of P2, P3 longer than propodus and carpus; merus with distinct longitudinal median groove on dorsal view in P2, merus with distinct median groove on lateral and dorsal face in P3 (Fig. 5E); P4 dactylus same in length as propodus and carpus, margins denticulate, merus relatively stout, about 4.0 times longer than wide, with distinct longitudinal median groove on latero-dorsal surface (Figs 4E, 8F).

Thoracic sternum wide (Figs 5F, 7D), surface densely packed with tubercles, sternites 1 and 2 fused, separated from sternite 3 by shallow groove; sternite 3 separated from sternite 4; sternites 4–7 well developed, separated from each other by well-developed suture; sternite 8 not exposed; sternopleonal cavity smooth, reaches base of sternite 3 (Figs 5H, 7D–E). Vulvae large, operculum cover present, located distally on bulged, sloping region of inner margin between sternites 6–8, on sternite 5; pleonal locking spine on sternite 4 (Figs 5H, 7D–F). Pleon wide, granulated, with 6 free somites, somite 3 widest; telson longer than wide, semicircular (Fig. 6J). Male unknown.

##### Colour.

Unknown.

##### Variation.

Males are unknown for this species, and the type females do not vary substantially among the known specimens.

##### Etymology.

The name of the species is derived from the Latin word *albucus*, bulb of the lily; alluding to the structure of the bulging vulva which is diagnostic of the species. The name is used as a noun in apposition.

##### Type locality.

Off Kochi, Kerala, Arabian Sea.

##### Distribution.

Presently known only from the type locality.

##### Remarks.

*Mariaplax albucus* sp. nov. differs from *M. sundaica* and *M. kochazhi* sp. nov. in that the subquadrate carapace, about 1.8 times as wide as long (Figs 4A, 5A, 6B, 8D) (versus the carapace about 1.7 times as long as wide in *M. sundaica*; Fig. 8G; see Rahayu and Widyastuti 2018: figs 9A, B, 10A; and 1.5–1.7 times as wide as long in *M. kochazhi*; Fig. 8A); the pterygostomial region is granulated with a row of six oblique striae *M. albucus* sp. nov. (Figs 4B, 5B, 8E) (versus with relatively reduced granulation and a row of four oblique striae in *M. sundaica*; Fig. 8H; Rahayu and Widyastuti 2018: figs 9B, F; and with 5–7 oblique striae with the upper margin weakly granulated in *M. kochazhi* sp. nov.; Figs 1C, 8B); the epistome is relatively longer, distally pointed, with the lateral margins distinctly bilobed (Figs 4B, 5B, 8E) (versus epistome relatively shorter, distally pointed and the lateral margins entire in *M. sundaica*; Fig. 8H; see Rahayu and Widyastuti 2018: fig. 9F; and being distally more pointed with weakly bilobed lateral margins in *M. kochazhi* sp. nov.; Figs 1C, 8B); the ischium of the third maxilliped as long as wide, more rectangular, with a more pronounced subproximal expansion and forming a triangular projection (Figs 5B, 6E, 8E) (versus the ischium of the third maxilliped slightly narrower, with the merus as long as wide in *M. sundaica*; Fig. 8H, see Rahayu and Widyastuti 2018: figs 9B; and the ischium slightly longer (0.7 times width) and more rectangular in shape, with more densely packed larger granules on the margins in *M. kochazhi* sp. nov.; Figs 1D, 6D, 8B); the P4 merus is comparatively longer, 4.0 times width with the median groove distinct on the lateral surface and visible in the dorsal surface, with densely packed granules, dactylus slightly wide and upcurved (Figs 4A, 5C, 8F) (versus P4 merus short, 3.4 times as long as wide, the longitudinal groove being absent on the lateral surface, dactylus wide, short, upcurved, spatuliform in *M. sundaica*; Fig. 8I, see Rahayu and Widyastuti 2018: figs 9A, 10H; and the P4 merus is short and stout, with deep grooves on lateral surface of the merus, densely packed granules, the dactylus being gently upcurved and narrow in *M. kochazhi* sp. nov.; Fig. 8C); and the vulva is comparatively large, with a well-developed operculum cover and positioned distally on the surface of the bulged, sloped margin of sternite 5 (Figs 5H, 7D–F) (versus vulva large, subovate in shape, operculum absent and positioned on the surface of slightly bulged, sloped distal margin of sternite 5 in *M. sundaica*; Rahayu and Widyastuti 2018: fig. 9E; and vulva is simple, rounded, completely exposed, with the operculum and the bulged sloping area absent in *M. kochazhi*; Figs 2C, 7A–C).

#### Genus Hexapinus

Taxon classificationAnimaliaDecapodaHexapodidae

Manning & Holthuis, 1981

4EABB201-7A10-567A-8B69-C964236A298F

##### Type species.

*Hexapinus latipes* (De Haan, 1835) (type species *Hexapus latipes* De Haan, 1835, by original designation; gender masculine).

##### Remarks.

The genus *Hexapinus* Manning & Holthuis, 1981 is represented by seven known species, only three species, *H. edwardsi* (Serène & Soh, 1976), *H. simplex* Rahayu & Ng, 2014, and *H. okinawa* Sato, Reimer & Naruse, 2025, possess a proportionately wider carapace and a granulated anterolateral margin with a bilobed angle on the posterolateral margin (see Rahayu and Ng 2014; Rahayu and Widyastuti 2018; Sato et al. 2025). We here recognise a fourth species from this group.

#### Hexapinus clivus sp. nov.

Taxon classificationAnimaliaDecapodaHexapodidae

164443B4-1626-568B-AF8F-147971421480

https://zoobank.org/82292E83-E65C-4732-A2B9-1FF11EAB6EB0

**Figure 9.**
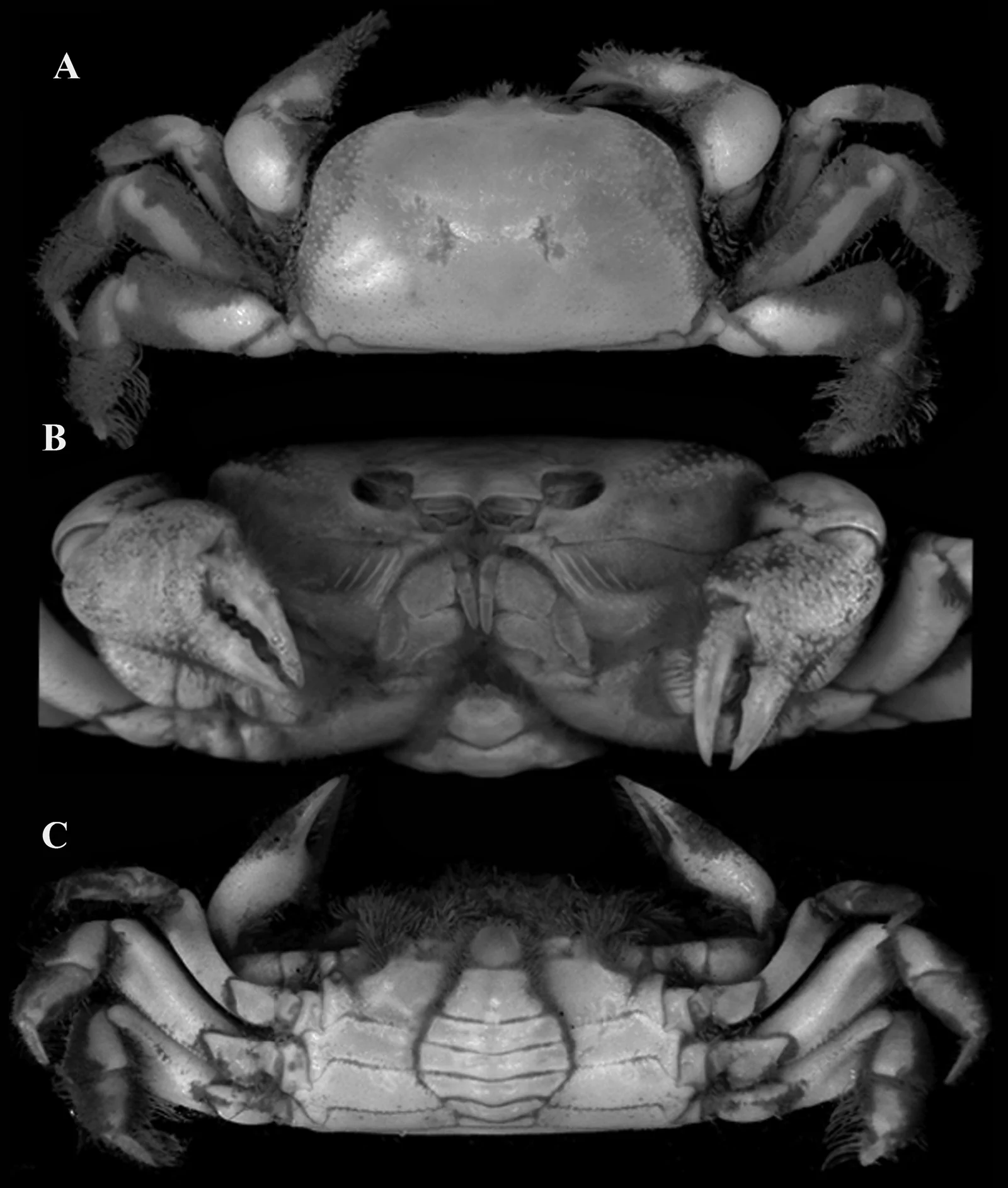
*Hexapinus clivus* sp. nov., holotype, female (8.8 × 5.6 mm) (CMLRE-BC-BRY-8), off Tamil Nadu, Bay of Bengal, India. **A**. Overall dorsal habitus; **B**. Frontal view of cephalothorax showing third maxillipeds and pterygostomial region; **C**. Ventral view of cephalothorax.

**Figure 10.**
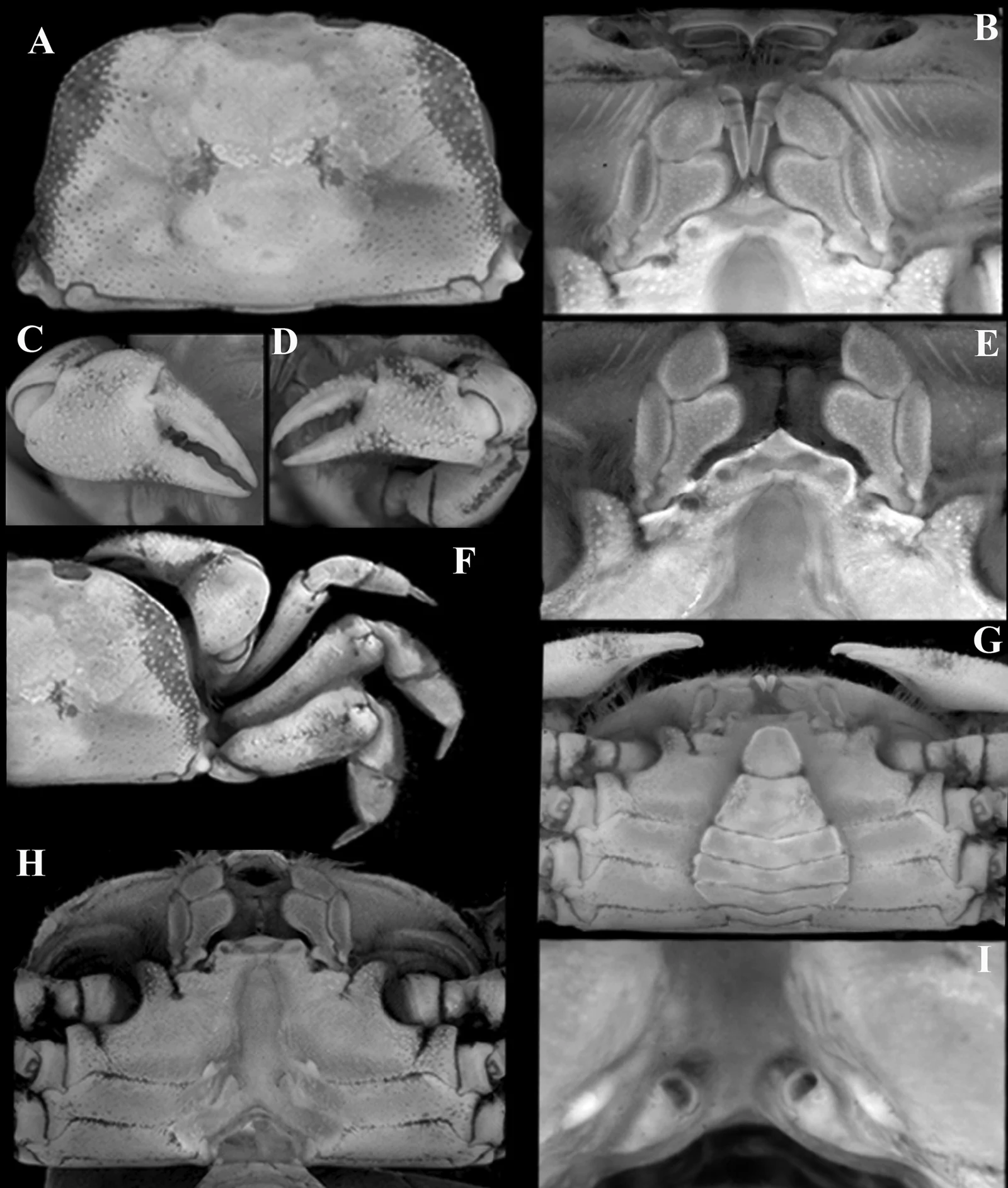
*Hexapinus clivus* sp. nov., holotype, female (8.8 × 5.6 mm) (CMLRE-BC-BRY-8), off Tamil Nadu, Bay of Bengal, India. **A**. Dorsal view of carapace; **B**. Frontal view of cephalothorax showing third maxillipeds, pterygostomial region and anterior thoracic sternum; **C**. Outer view of right major chela; **D**. Outer view of left minor chela; **E**. Third maxillipeds and anterior thoracic sternum; **F**. Dorsal view of ambulatory legs and chelae; **G**. Thoracic sternum and pleon; **H**. Thoracic sternum and sternopleonal cavity; **I**. Vulvae.

**Figure 11.**
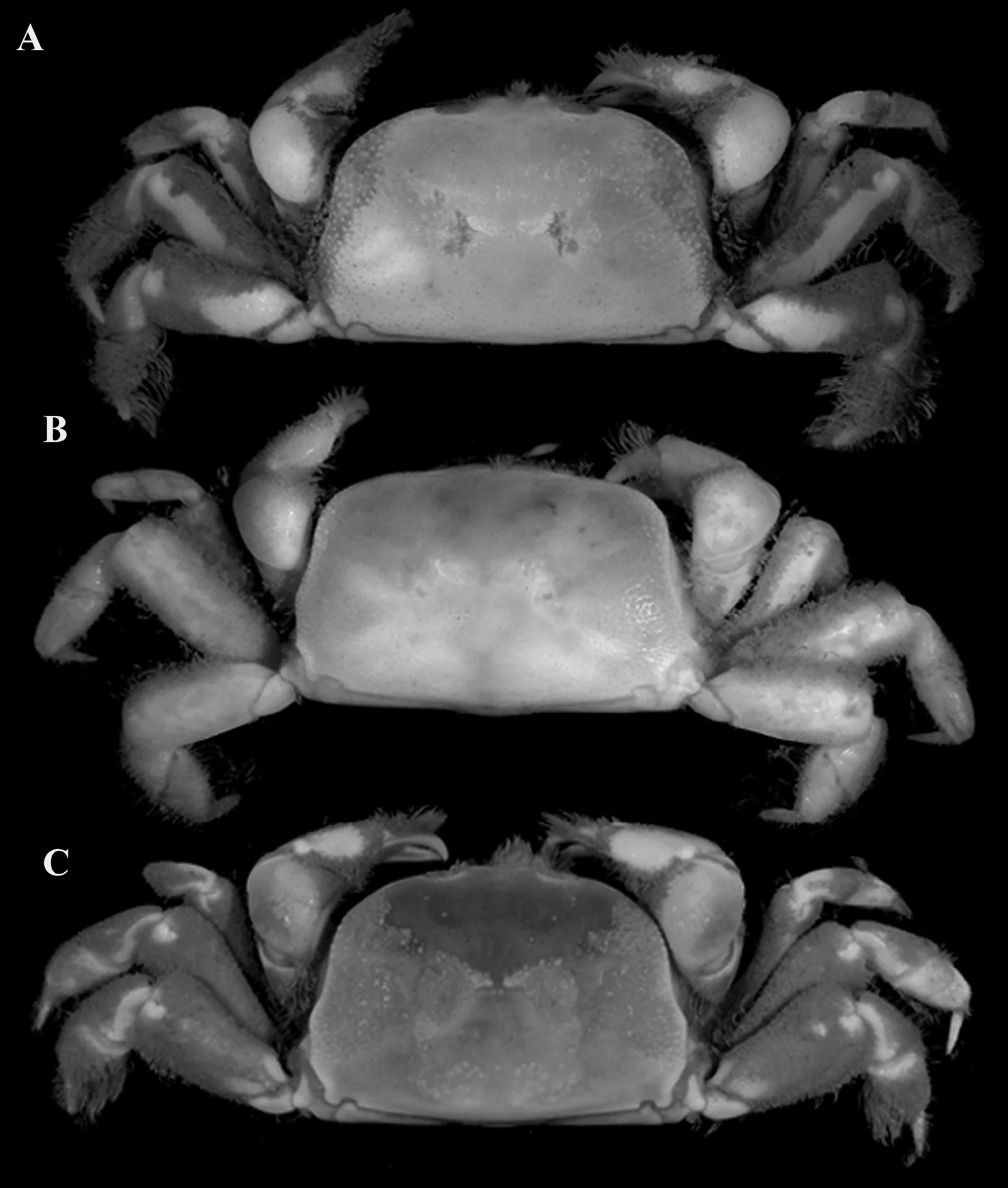
Overall dorsal habitus. **A**. *Hexapinus clivus* sp. nov., holotype female (8.8 × 5.6 mm) (CMLRE-BC-BRY-8), off Tamil Nadu, Bay of Bengal, India; **B**. *H. simplex*, male (4.5 × 3.3 mm) (ZRC 2014.0224), Pulau Tekong east and Pengerang, Singapore; **C**. *H. okinawa*, holotype female (12.0 × 17.6 mm) (RUMF-ZC-8244), Okinawa Island, Japan [photographed by Tohru Naruse].

**Figure 12.**
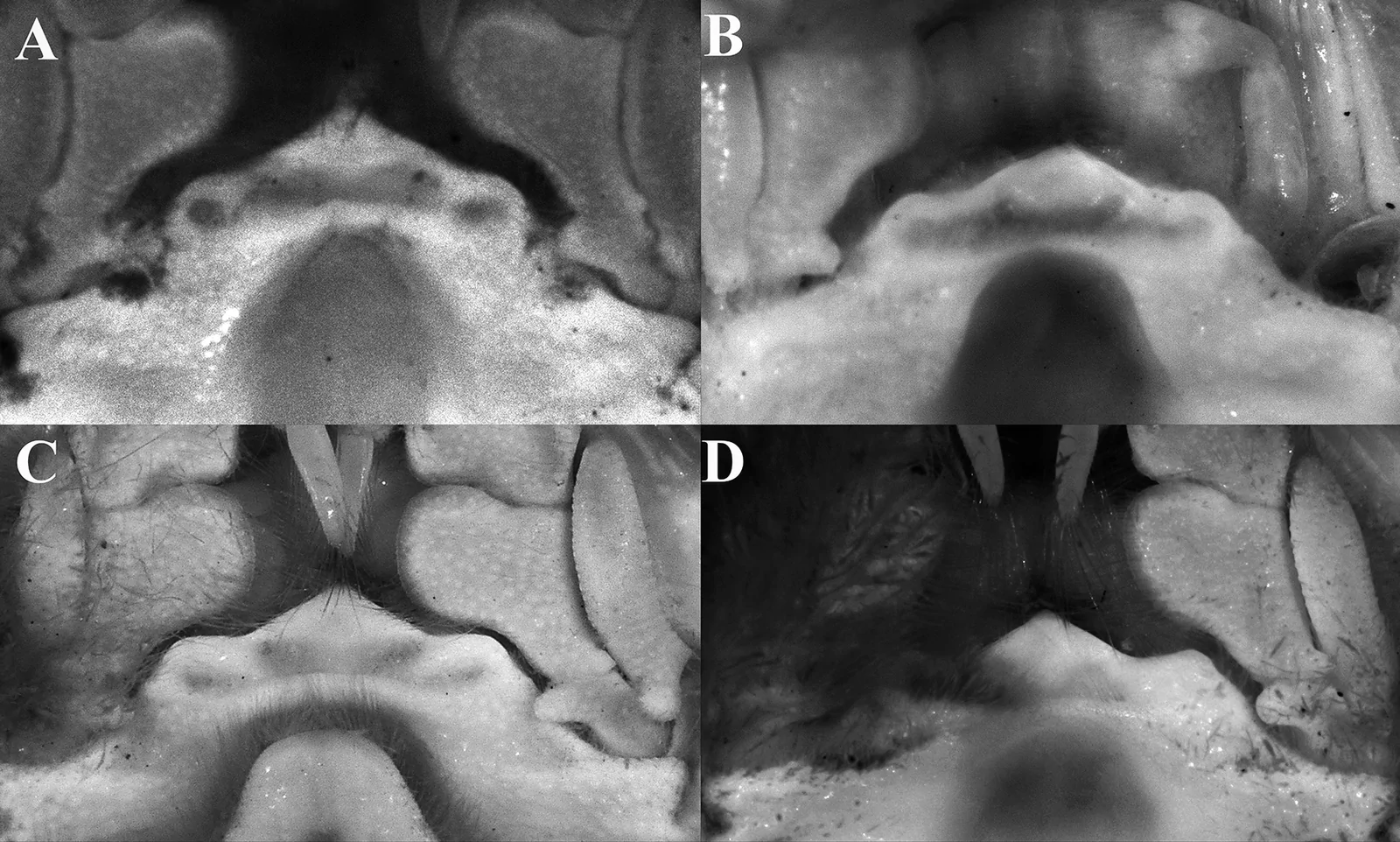
Thoracic sternites 1–3. **A**. *Hexapinus clivus* sp. nov., holotype female (8.8 × 5.6 mm) (CMLRE-BC-BRY-8), Bay of Bengal; **B**. *H. simplex*, female (10.3 × 9.0 mm) (ZRC 2012.1016), Taiwan Strait; **C**. *H. simplex*, male (9.3 × 5.8 mm) (ZRC 2014. 0203), Sulawesi; **D**. *H. okinawa*, holotype female (12.0 × 17.6 mm) (RUMF-ZC-8244), Okinawa [photographed by Tohru Naruse].

**Figure 13.**
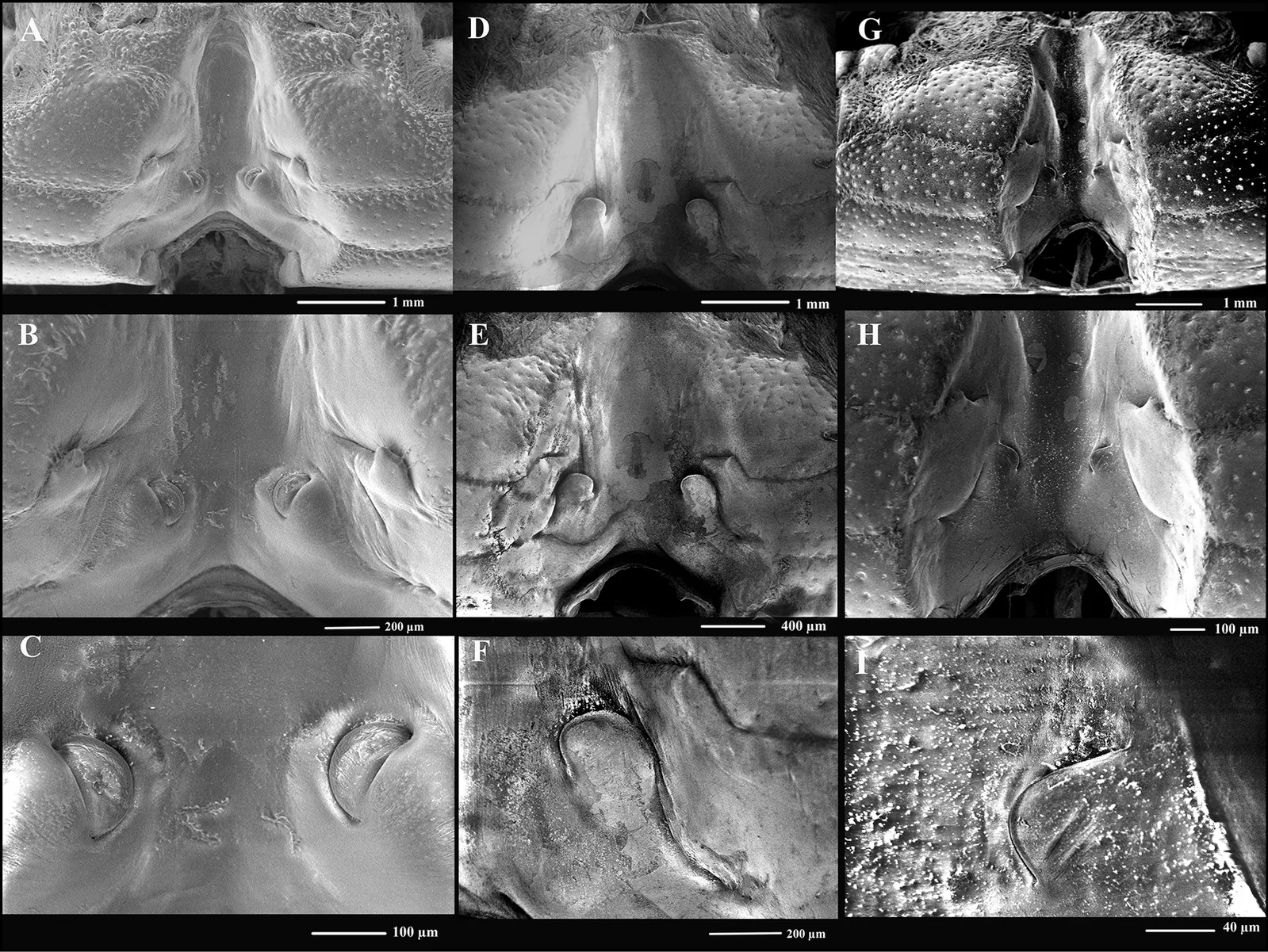
SEM micrographs of female thoracic sternum and vulvae. **A–C**. *Hexapinus clivus* sp. nov., holotype female (8.8 × 5.6 mm) (CMLRE-BC-BRY-8), Bay of Bengal; **D–F**. *H. simplex*, paratype, 1 adult female (10.1 × 5.8 mm) (CMLRE-BC-BRY-10, ex. ZRC 2010.0297), Lombok; **G–I**. Juvenile female (6.9 × 3.8 mm), same data as previous; **A, D, G**. Overall view of sternopleonal cavity; **B, E, H**. Close-up view of sternites 4**–**6 and vulvae; **C**. Close-up view of adult vulvae; **F**. Close-up view of adult left vulva; **I**. Close-up view of left vulvae of juvenile female.

Figs 6C, 6F, 6J, 9, 10, 11A, 12A, 13A–C

##### Material examined.

***Holotype***. • 1 female (8.8 × 5.6 mm) (CMLRE-BC-BRY-8), station 11, cruise number 392, dredge, 433 m, sandy bottom, off Tamil Nadu, Bay of Bengal 11°57'37.8"N, 80°09'13.7"E, coll. S.S. Cubelio, 29 November 2019. ***Paratype***. • 1 juv. female (6.7 × 4.6 mm) (ZRC 2019.1840), from trawl bycatch, Shankarpur coast, West Bengal, India, coll. M. Dev Roy, March 2018.

##### Comparative material.

***Hexapinus simplex* Rahayu & Ng, 2014**. **Indonesia: *Paratypes***. • 2 males (8.6 × 5.1 mm, 5.5 × 3.5 mm) (CMLRE-BC-BRY-9); • 2 females (10.1 × 5.8 mm, 6.9 × 3.8 mm), (CMLRE-BC-BRY-10, ex. ZRC 2010.0297), Ekas, Eastern Lombok, 8°52'09"5N, 116°27'26.1"E; • 1 male (9.3 × 5.8 mm) (ZRC 2014.0203), Sulawesi, Lembeh Straits. coll. H.H. Tan, 17 July 2023; **Singapore**: • 1 female (10.2 × 5.7 mm) (ZRC 2013.1686), St. John Island, north lagoon, 1°13.116'N, 103°51.079'E, coll. P.K.L Ng and A. Anker, 31 May 2013; • 1 ovigerous female (8.0 × 4.5 mm) (ZRC 2013.1687), DR2, near Raffles Lighthouse, R.V. Galaxea, 20 May 2013; • 1 male (9.1 × 5.7 mm) (ZRC 2014.0227), stn. SEN–AA04, Pulau Senang, north side near jetty, intertidal sand-mud flat with abundant rubble and rocks, from burrow together with chaetopterid worm, 0.5 m, yabby pump, 01°10.504'N, 103°44.1790'E, A. Anker, 30 March 2014; • 1 male (4.5 × 3.3 mm) (ZRC 2014.0224), station. 0523 DR3, between Pulau Tekong east and Pengerang (Malaysia), 10.6–10.7 m, trawl, sandy with broken shell substrate, 01°23.914'N – 01°24.141'N, 104°05.353'E – 104°05.400'E, coll. S.C. Lim et al. 25 March 2014; **China**: • 1 male (10.3 × 6.8 mm), 1 female (10.3 × 9.0 mm) (ZRC 2012.1016), Taiwan Strait, Fujian Province, Dongshan, pearl shell culture ground, station 75C–2368, 1 Jun.1975; • 1 female (11.4 × 9.8 mm) (ZRC 2012.1017), southeast Hainan Island, station 6160, 18°30'N, 110°30'E, South China Sea, coll. D.L. Rahayu; 1 September 2013; **Hong Kong**: • 1 male (9.1 × 5.6 mm), 1 female (10.3 × 6.7 mm) (ZRC 2019.1102), from *Chaetopterus* tubes, J.W Qiu, 20 April 2015; **Japan**: • 2 females (11.3 × 8.8 mm, 10.3 × 8.6 mm) (ZRC 2012.1018), Takebu Amami Islands, 25 April. 2012; • 1 male (9.1 × 8.7 mm) (ZRC 2013.1688), Iriomote Island, Funaura Bay, coll. T. Naruse, 1 July 2011. ***Hexapinus okinawa* Sato, Reimer & Naruse, 2025. Japan: *Holotype***. • female (12.0 × 17.6 mm) (RUMF-ZC-8244), Red Beach, Kin Bay, Okinawa Island, Ryukyu Islands, Japan, 26.446043°N, 127.911707°E, ca. 10 m, SCUBA and yabbie pump from *Chaetopterus* tube, coll. T. Sato and H. Nakajima, 16 April 2022 [photographs examined].

##### Diagnosis.

Carapace subquadrate, region indistinct, surface smooth; antero-posterolateral margin protuberance bilobed, densely packed with granules; pterygostomial region with a row of 4 oblique striae, entire; thoracic sternum wide, sternites 1 and 2 fused, subtriangular, laterally forming gentle slope, separated from sternite 3 by distinct curved lateral margin; pleon subovate, all somites free; vulva semilunate, oblique with elevated protrusion. P2–P4 short, stout, P3 longest; P4 merus about 2.5 times as long as wide.

##### Description.

Carapace subquadrate, 1.6 times as wide as long, dorsal surface smooth, region indistinct, median H-shaped depression visible; larger granules present on anterolateral margins, only pitted surface on posterior margin (Figs 9A, 10A); anterolateral margin entire, arcuate, posterolateral margin subtrapezoidal protuberance with bilobed lateral margin, densely packed with granules; posterior margin weakly convex ventrally, lateral ends concave, accommodating small condyle of thoracic sternite 7 (Figs 9A, 10A, 11A); frontal margin deflexed, establishing triangular lobe in frontal view, forming part of interantennular septum, medially concave, surface smooth; eyes slender, small, slightly movable, pigmented cornea, supra- and infraorbital margins smooth, entire, mesial end of infraorbital margin upwardly produced as subtriangular inner orbital tooth; suborbital and subhepatic regions with granules; pterygostomial region with a row of 4 oblique striae (Fig. 10B); branchial region covered with very short, dense setae (Fig. 9C). Third maxillipeds relatively wide, densely granulated, slightly setose, ischium 1.5 times as wide as long, slightly longer than merus, strongly expanded distomesially, with rounded mesial margin; combined length of dactylus, propodus and carpus shorter than that of merus and ischium; combined length of dactylus, propodus and carpus shorter than that of merus and ischium; flagellum well developed (Figs 9B, 10B, 10E). Chelipeds stout, unequal (Fig. 9), dactylus with tufts of setae on upper and lower margins proximally; major chela; slightly gaping when fingers closed; palm slightly wider than long, outer surface densely covered with tubercles; minor chela with wider gap between closed fingers (Fig. 10C, D). P2–P4 (Fig. 9A, C) short, stout, P3 longest; P4 merus about 2.5 times as long as wide; dactylus of P2–P4 straight (Fig. 10F).

Thoracic sternum wide, sparsely pitted medially, and tuberculated marginally (Figs 9C, 10G–I, 12A, 13A–C), sternites 1 and 2 fused, subtriangular, pointed towards buccal cavity, slightly concave medially, separated from sternite 3 by distinct curved lateral margin; lateral margin of fused sternites 1 and 2 gradually joining that of sternite 3, forming gentle curve; sternite 3 separated from sternite 4 laterally, medially shallow groove, extends small transverse ridges from sternoabdominal cavity to suture of sternites 3 and 4 (Fig. 13A–C); sternites 4–7 very wide, separated from other somites by distinct granulated sutures, medially appears triangular projection; sternite 8 invisible; sternopleonal cavity subovate, smooth, anterior end spatulate, reaches base of sternite 2 (Figs 10B, 10E, 10H, 13A–C); sternopleonal locking spine well developed, hooked structure on suture 4/5, appears close to vulva; vulva located on suture 5/6, below to locking spine, medially closure, elevated protrusion, semilunate, oblique (Fig. 13A–C).

Pleon subovate, all somites free, lateral margins covered with long setae; somites 3, 4 widest; laterally somite 3 slightly concave, somite 4 straight; somite 6 longest, subtrapezoidal, convergent distally, lateral margin weakly concave; telson subovate, shorter than somite 6 (Fig. 9C, G).

##### Colour.

Unknown.

##### Variation.

The small paratype female (ZRC 2019.1840) is similar to the holotype female (CMLRE-BC-BRY-8) except that its pleon is less expanded, the pleopods are poorly developed, and the vulvae do not appear mature.

##### Etymology.

The species name is derived from the Latin word *clivus*, sloping side of a mountain; alluding to the sides of the triangular structure of thoracic sternites 1 and 2, which is a diagnostic character of the species. The name is used as a noun in apposition.

##### Type locality.

Off, Kochi, Kerala, Arabian Sea.

##### Distribution.

Presently known only from the type locality.

##### Remarks.

The recognition of the present new species is connected to the identity of *Hexapus edwardsi*, which was described from a single small subadult male 4.8 × 3.0 mm from the Andaman Sea. Rahayu and Ng (2014: 409) transferred the species to *Hexapinus* based on the structure of the third maxilliped as well as the form of the male pleon. The male pleon as figured by Serène and Soh (1976: 25, fig. 27B) is unusual because somite 5 seems separate from fused somites 3 and 4 (somite 1 apparently not figured). In typical *Hexapinus*, male pleonal somites 3–5 are fused with somite 6 elongate (e.g., see Rahayu and Ng 2014: fig. 21E–G). Even in small subadult males, this is apparent (Rahayu and Ng 2014: fig. 21E). Females, on the other hand, have all the somites free, so it seems unlikely Serène and Soh’s (1976) type specimen is a female because at least somites 3 and 4 are fused. Whether the figure in Serène and Soh (1976) is accurate will require re-examination of the specimen. Serène and Soh (1976: 25) described the anterolateral margin of *H. edwardsi* as “like an open continuous arch; the lateral borders converge regularly forward and are not parallel” and “the antero-lateral region and the external surface of the palm of chelipeds are ornamented with a covering of acute granules, disappearing under a short tomentum”. This description generally matches that of *Hexapinus clivus* sp. nov., except that its carapace appears somewhat more rectangular with the anterolateral margins more angular (Figs 9A, 10A) whereas in *H. edwardsi*, the carapace is more semicircular with the anterolateral margin clearly more rounded (cf. Serène and Soh 1976: pl. 7D). In addition, the merus of the third maxilliped is distinctly more quadrate and longer (Figs 6F, 10B, 10E) than that of *H. edwardsi* (cf. Serène and Soh 1976: fig. 27A). *Hexapinus clivus* sp. nov. is clearly close to *H. edwardsi* but the differences noted here indicate that we should treat them as different species for the time being.

The characters of the present new species agree best with two West Pacific species: *H. simplex* Rahayu & Ng, 2014 (from East and Southeast Asia) and *H. okinawa* Sato, Reimer & Naruse, 2025 (from the Ryukyu Islands), but it can be clearly separated by a suite of distinct characters. *Hexapinus clivus* sp. nov. is distinguished with its subquadrate carapace, as wide as long, with lateral margins moderately curved and densely granulated (Figs 9A, 10A, 11A) (versus carapace elongated, lateral margins slightly curved and granulated in *H. simplex*; Fig. 11B; with carapace wider, lateral margin deeply curved and moderately granulated in *H. okinawa*; Fig. 11C); the third maxilliped ischium as wide as long and is strongly expanded distomesially with a rounded mesial margin (Figs 6F, 10B, 10E) (versus third maxilliped ischium 1.1 times as wide as long, slightly shorter in *H. simplex*; Fig. 12B; and ischium 0.7 times as long as ischium and much shorter in *H. okinawa*; Fig. 12D); the lateral margin of fused female thoracic sternites 1 and 2 gradually joining lateral margin of sternite 3 forming a gentle curve (Fig. 12A) (versus lateral margin of fused sternites 1 and 2 joining that of sternite 3 at a distinct obtuse angle in *H. simplex*; Fig. 12B, C; and *H. okinawa*; Fig. 12D); P3 much longer than P4, lower margin with serrated granules, and P4 about as long as wide (Figs 9A, 9C, 10F, 11A) (versus P3 slightly longer than P4, lower margin smooth, P4 shorter in *H. simplex*; Fig. 11B; and P3 slightly longer than P4, lower margin with serrated granules, with P4 short and wide in *H. okinawa*; Fig. 11C); and the vulva semilunate with elevated protrusion, medially closed, with a well-developed, hook-like locking spine (Fig. 13A–C) (versus vulva subovate without protrusion, medially wider, and locking spine not hook-like in adult *H. simplex*; Fig. 13D–F; with the condition in juveniles subovate, not fully developed with the locking spine weakly hooked; Fig. 13G, H; and vulva elliptical, with posterolateral margin elevated to form hood-like protrusion in *H. okinawa*; Sato et al. 2025: fig. 1D).

## Supplementary Material

XML Treatment for Mariaplax

XML Treatment for Mariaplax kochazhi

XML Treatment for Mariaplax albucus

XML Treatment for Hexapinus

XML Treatment for Hexapinus clivus

## Additional information

### Artificial Intelligence (AI) use

The authors accept full responsibility for the content of the manuscript, including the disclosure of any use of AI.

No AI tools were used in the preparation of this manuscript.

### Author ORCIDs

M. Prema https://orcid.org/0000-0003-2694-3034

S.S. Cubelio https://orcid.org/0000-0002-2960-7055

P.K.L. Ng https://orcid.org/0000-0001-5946-0608

### Data availability

All of the data that support the findings of this study are available in the main text.
